# Exploring the Effects of Acute Stress Exposure on Lumpfish Plasma and Liver Biomarkers

**DOI:** 10.3390/ani13233623

**Published:** 2023-11-23

**Authors:** Tiago da Santa Lopes, Benjamin Costas, Lourenço Ramos-Pinto, Patrick Reynolds, Albert K. D. Imsland, Jorge M. O. Fernandes

**Affiliations:** 1 Gildeskål Forskningsstasjon AS, 8140 Inndyr, Norway; tiago@gifas.no (T.d.S.L.); pat.reynolds@gifas.no (P.R.); 2 Faculty of Biosciences and Aquaculture, Nord University, 8026 Bodø, Norway; 3 Interdisciplinary Centre of Marine and Environmental Research (CIIMAR), 4450-208 Matosinhos, Portugal; bcostas@ciimar.up.pt (B.C.); lourenco.pinto@ciimar.up.pt (L.R.-P.); 4 Instituto de Ciências Biomédicas Abel Salazar (ICBAS-UP), Universidade do Porto, 4050-313 Porto, Portugal; 5 Department of Biological Sciences, University of Bergen, 5020 Bergen, Norway; aki@akvaplan.niva.no; 6 Akvaplan-niva Iceland Office, 201 Kópavogur, Iceland

**Keywords:** cleaner fish, lumpfish, salmon farming, stress, health, welfare

## Abstract

**Simple Summary:**

Farmed fish are commonly exposed to stressors, which have a detrimental effect on their health and welfare. The present study explores how lumpfish, a species recognized for its potential role in alleviating the sea lice burden on farmed salmon, responds to acute stress. This is particularly important for this species, since there are concerning reports regarding their health and welfare. In our stress challenge lumpfish were exposed to air for one minute. We then examined several stress and energy metabolism biomarkers and concluded that i) lumpfish tolerates well a one-minute air exposure, and ii) cortisol is a reliable stress biomarker in this species. Our results contribute to our current understanding of how lumpfish handle a common stressor, and this knowledge is key to allow a better decision-making when it comes to welfare management in farms.

**Abstract:**

This study aimed to expand knowledge on lumpfish stress physiology by investigating the effects of acute stress on primary (i.e., cortisol) and secondary (e.g., metabolites) stress responses, as well as oxidative stress biomarkers, from stress exposure to a recovery phase. The results showed that the lumpfish physiological response to 1 min air exposure is mild, in line with recent studies, and comparable to that described for white sturgeons. Cortisol seems to be the most reliable acute stress biomarker in lumpfish, with a significant increase in plasma 30 min after stress exposure, returning to resting levels 2 h after exposure. In contrast, glucose and lactate were not significantly altered by short-term air exposure. Effects on hepatic energy mobilisation were also detected following the acute stress. This study showed that acute 1 min air exposure seems tolerable, allowing a swift recovery. However, more studies on the impacts of air exposure and repeated acute stressors on lumpfish stress and immune responses are required to develop industry standards for lumpfish health and welfare monitoring.

## 1. Introduction

One of the main constraints that Atlantic salmon (*Salmo salar*) aquaculture faces is the persistent ectoparasite infestation caused by sea lice, in particular, *Lepeoptheirus salmonis* and other Caligidae member species [1,2]. In response to the persistent impact of sea lice, causing an estimated economic impact of more than USD 500 million [3], research has increasingly focused on treatment, prevention and alternative delousing methods [4,5,6]. Lumpfish (*Cyclopterus lumpus,* L.) is a species of interest in the salmon aquaculture sector, due to its role in the biological control of sea lice. As such, lumpfish aquaculture gained traction in recent years as a reaction to this urgent need for efficient delousing alternatives [7,8,9,10]. In fact, lumpfish individuals deployed in salmon pens increased from approximately 10 million in 2015 to more than 40 million in 2019 [11]. This rapid development has led to an increasing amount of concerning evidence regarding the health and welfare of these animals under farming operations [12]. A recent report by the Norwegian Food Authorities, based on a 2018/2019 campaign [13] on the use of lumpfish by Norwegian Atlantic salmon farmers, presented disturbing data regarding the mortality and fate of deployed lumpfish. In this campaign, farmers reported mortality above 40%, often associated with disease outbreaks. Lumpfish are exposed to several stressful events under farming operations, from hatchery rearing to transportation and deployment in salmon sea pens [14,15,16]. It is well known that stress significantly influences the immune status in teleosts [17,18]. Accordingly, the acute and chronic stress experienced by lumpfish could contribute to the frequently reported disease outbreaks and high mortalities [19,20,21,22]. Given the current limited understanding of lumpfish biology, optimal rearing conditions, dietary needs and welfare monitoring, it is crucial to direct scientific efforts towards expanding knowledge in these areas [20,23,24].

Organisms employ various strategies to regain homeostasis by regulating physiological processes that compensate for, adjust to, and mitigate stress. An organism’s ability to cope with stress and regain homeostasis depends on the severity of the stressor. When the severity of the stressor pushes the organism’s ability to cope with the stress over a critical limit, physiological mechanisms are disrupted, welfare condition deteriorates and ultimately, the organism succumbs [25,26].

Teleost fish have evolved a stress response that can be categorised into three different levels. A primary response, which starts with the detection of the stressor, triggers an immediate neuroendocrine cascade of events that results in the release of catecholamines and cortisol. A secondary response to stress is coordinated by the primary response hormones, where energy mobilisation is regulated through metabolic and osmoregulatory adjustments, to meet the increased energy demands to fuel the stress response. The tertiary response is an outcome of the inability of an organism to regain homeostasis and is characterised by changes and impairments at the systemic level, such as suppressed immunity and reduced growth [27,28,29].

The primary stress response in teleosts begins with activating the sympatho-chromaffin tissue (SC axis) and the hypothalamus–pituitary–interrenal tissue (HPI axis) [30]. The SC axis is quickly mobilised upon perceiving a stressor, increasing heart rate, stroke volume, blood flow and increasing glucose supply to critical tissues [29,31,32]. Subsequently, the HPI axis is activated. The central nervous system stimulates neurons in the hypothalamus to produce a corticotropin-releasing hormone (CRH), leading to the release of corticotropin (ACTH), which in turn will stimulate the production of cortisol, by the interrenal cells. Cortisol and catecholamines play pivotal roles in orchestrating the subsequent stages of the stress response. These hormonal responses primarily prepare the organism for increased energy demands during the “fight or flight” response, where glucose serves as the primary energy substrate in the brain and muscle [29]. This energy cost is met, initially, by the activation of the SC axis, releasing epinephrine in seconds, and sustained by the activation of the HPI axis, which results in the release of cortisol, inducing and maintaining a hyperglycaemic state [32,33,34]. Cortisol regulates blood glucose levels, sustains hyperglycaemia and influences the mobilisation of amino acids, fatty acids and lactate for gluconeogenesis [35].

Acute stressors are not rare during aquaculture operations involving lumpfish, which shows the urgent need for stress monitoring. From being introduced into net pens with Atlantic salmon without prior interaction, or transportation, to the grading of salmon and mechanical delousing, lumpfish are often handled and exposed to stressors, which usually involve temporary air exposure [16,19,36]. These highly stressful conditions such as hypoxia, handling and osmotic stress can induce marked metabolic variations. Besides cortisol, lactate and glucose concentrations can change significantly upon stressful events and are often used as indicators of primary and secondary stress responses [37,38,39]. Oxidative damage parameters such as lipid peroxidation also display the potential to be used as stress biomarkers. In healthy individuals, the antioxidant defences neutralise the reactive oxygen species (ROS) produced from regular metabolic activity, maintaining an optimal equilibrium [40,41,42,43]. However, stress can disrupt the oxidative balance by inducing a higher metabolic turnover, increasing ROS and causing oxidative damage [42,43,44,45]. The lumpfish, a peculiar species, exhibits a mild increase in cortisol and other stress biomarkers upon acute stress exposure. This subtle response can often be misunderstood as an absence of stress, potentially unnoticed by salmon farmers and lumpfish hatcheries. Stress studies with lumpfish have identified modest cortisol, glucose and lactate increases post-stress, comparable to several sturgeon species [36,46,47]. Understanding the metabolic changes upon acute stress in lumpfish is paramount and will allow the development of early detection tools as well as the accurate monitoring of lumpfish stress status. This study aimed to understand the influence of brief air exposure on lumpfish metabolism and stress markers.

## 2. Materials and Methods

### 2.1. Fish Acquisition and Aare

Juvenile lumpfish with a mean weight of 70.0 g were transferred from Artic Seafood Group (Mørkvedbukta, Bodø, Norway) and maintained for 2 weeks in indoor 1000 L flow-through seawater tanks at Mørkvedbukta research station (Nord University, Mørkvedbukta, Norway), using a 12 h light–dark photoperiod. Water parameters were checked daily, including salinity (34.6 ± 0.5‰), dissolved oxygen (7.9 ± 0.3 mg L^−1^) and water temperature (9.4 ± 0.6 °C). The health status of the fish was assessed immediately after transfer using the Lumpfish Health Scoring System guidelines [19]. Lumpfish in all tanks were fed a commercial lumpfish feed (Skretting’s Clean Assist, 1.8 mm pellet size, Skretting, Stavanger, Norway). The feeding regime was based on 2% BW per day using mechanical feeding automats, with 6 meals throughout the day.

### 2.2. Study Design

Ten duplicate groups of lumpfish with an initial mean (± SD) weight of 70.0 ± 5.0 g (*n* = 16; N = 160) were established from the original population (groups 30minS, 30minC, 1hS, 1hC, 2hS, 2hC, 4hS, 4hC, 24hS, 24hC). Numbers in the group name indicate the time of sampling (in min or hours) after the start of the experiment. The letter “S” indicates groups exposed to stress, and the letter “C” indicates control groups, where fish were left undisturbed until sampling. Each fish group was kept in a separate tank. The fish were allowed to acclimate for a period of 2 weeks before the start of the trial, when they started exhibiting normal swimming and hovering behaviour as well as a good feeding response. The trial commenced with the induction of stress (1 min air exposure) of “S” groups. To allow for better time management for the collection of samples, group 30minS was exposed to stress before the remaining groups. One day before the stress challenge and on the day of this challenge fish were not fed, to avoid the influence of feeding on the stress biomarker levels [48].

**Figure 1:**
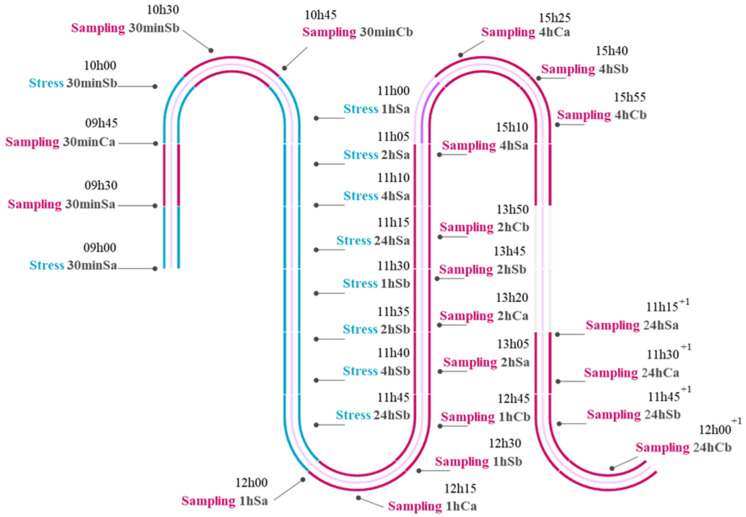
Diagram of the acute stress trial. Timeline of the 1 min air exposures (“Stress”) and sampling of each tank. “S” stands for stress group, “C” represents control (undisturbed) groups and “a” and “b” stand for each replicate within each group.

Fish from S groups (“S”, Stressed) were air-exposed for 1 min using a large net. Catching the fish with the net lasted less than 30 s in each tank. To ensure adequate sampling time, we stressed each duplicate tank from the same group 30 min after the air exposure of the first duplicate tank (Figure 1). The group 30minS was the exception, which had a 1 h window between replicate stress exposures to ensure suitable time management for sampling and processing of samples. Groups 1hS, 2hS, 4hS and 24hS were subsequently air-exposed (with 30 min intervals between duplicates, as mentioned above).

### 2.3. Blood and Liver Sampling and Tissue Preparation

For sampling, all individuals were quickly removed from each tank at a time and euthanised with 1600 mg L^−1^ metacaine (Tricaine methanesulphonate, Sigma Aldrich Co, St. Louis, MI, USA) until fish reached stage IV of anaesthesia, with no detectable operculum movement. Blood was taken from the caudal vein using 21-gauge needles and 4 mL heparinised vacutainers.

Blood collection lasted less than 3 min to avoid a cortisol increase due to manipulation during sampling. Blood was centrifuged (3000 rpm for 5 min at 4 °C) to obtain plasma, which was transferred to 1.8 mL cryotubes, snap-frozen in liquid nitrogen and stored at −80 °C until the analysis was performed.

Liver tissues were taken using sterilised steel scalpels and immediately transferred to two aliquots, in two separate 1.8 mL cryotubes to allow the preparation of liver samples for both oxidative stress and metabolite analyses, which require different homogenisation procedures. Liver samples were immediately snap-frozen in liquid nitrogen and stored at −80 °C.

Homogenisation of liver samples was necessary for metabolite and oxidative stress analyses. For metabolite analysis, the frozen liver was finely minced in a 50 mL Falcon tube, mixed and homogenised by mechanical disruption using a high-performance dispersing instrument (SilentCrusher M, Heidolph Instruments, Schwabach, Germany) in 7.5 vol. ice-cold 6% (*w*/*v*) perchloric acid. The homogenate was then neutralised with an equal volume of 1M KHCO_3_ and centrifuged (13,000× *g* for 30 min at 4 °C). Before centrifugation, aliquots of each homogenate were separated for the measurement of triglycerides and lactate. The remaining homogenates were then centrifuged (30 min, 13,000× *g*, 4 °C), and the supernatants were recovered in different aliquots and stored at −80 °C.

Homogenisation of frozen liver samples was also performed for oxidative stress assays, using a high-performance dispersing instrument (SilentCrusher M, Heidolph Instruments, Schwabach, Germany), in 1:10 volume K phosphate buffer (KPB) (K_2_HPO_4_ 0.1 M, KH_2_PO_4_ 0.1 M, pH 7.4, Sigma Aldrich, St. Louis, MI, USA). Two hundred microlitres of homogenate aliquots were used to measure lipid peroxidation. To prevent lipid peroxidation, 4 µL of 4% 3,5-di-tert-4-butylhydroxytoluene (BHT, in methanol, Sigma Aldrich, St. Louis, MI, USA) was added to each sample before centrifugation, making the aliquot for the lipid peroxidation assay, following the protocol by Torres et al. [49]. The homogenates were then centrifuged at 10,000× *g* for 20 min and 4 °C. The supernatants were kept in separate aliquots at −80 °C until later use.

### 2.4. Plasma and Liver Parameters

Plasma cortisol was measured using a commercial ELISA kit (IBL International GMBH, Hamburg, Germany). Plasma was diluted (1:20) in diethyl ether. After centrifugation, the recovered supernatant was isolated, and once evaporated, phosphate buffer containing 1 g.L^−1^ gelatine (pH 7.6) was added. This kit was previously validated for teleosts (Oliveira et al. [50]). We performed two tests to validate the lumpfish plasma samples: dilution parallelism and recovery. The dilution parallelism test consisted of 4 consecutive dilutions of a lumpfish plasma sample with a high concentration of cortisol, which was then compared with the standard curve. The curve obtained from lumpfish plasma was parallel to the standard curve, thus validating the test for this species (results not shown). The recovery test consisted of adding increasing amounts of cortisol to a lumpfish plasma sample, using the standards of the kit (standards D, E and F). A recovery value of 83.9 ± 13.6% was obtained, reinforcing the validity of the kit for this species. The main cross-reactivity (>1%, given by the supplier) was 30% for prednisolone, 11% for 11-Desoxy-Cortisol, 4.2% for cortisone, 2.5% for prednisone and 1.4% for corticosterone. Since cortisol is the principal steroid produced by fish interrenal tissue, cross-reactivity with other steroids was assumed to be negligible.

Plasma glucose, lactate and triglycerides, as well as hepatic lactate and triglycerides, were determined using Spinreact kits (Spinreact, Girona, Spain) adapted to 96-well microplates [39]. Plasma total proteins were determined in 1:50 (*v*/*v*) diluted plasma samples using Pierce™ BCA Protein Assay Kit (Thermo Fisher Scientific, Waltham, MA, USA), as reported by Costas et al. [51]. Bovine serum albumin (Thermo Fisher Scientific, Waltham, MA, USA) served as a standard. These parameters were analysed on a Synergy HT Microplate Reader (BioTek Instruments, Winooski, VT, USA).

### 2.5. Liver Oxidative Stress Assays

Liver samples were thawed and homogenised (1:10) in phosphate buffer 0.1 M (pH 7.4) using Precellys evolution tissue lyser homogeniser (Bertin Instruments, Montigny-le-Bretonneux, France).

Oxidative stress was investigated through enzymatic activity. Lipid peroxidation (LPO), superoxide dismutase (SOD) and catalase (CAT) activities, as well as total protein concentration, were quantified in the homogenised samples. CAT was determined by measuring the decline of H_2_O_2_ concentration as described by Clairborne (1985) [52] and adapted by Peixoto et al. [53]. Enzyme activity is expressed as enzyme units per milligram of total protein (U mg^−1^ protein). SOD was measured using the protocol described by Lima et al. [54] and adapted by Almeida et al. [55], where enzyme activity is calculated based on the amount of enzyme necessary to inhibit 50% of cytochrome c reduction rate, which occurs when superoxide radicals are present. Lipid peroxidation was measured using thiobarbituric acid-reactive substances (TBARSs) [49,56]. Total proteins in liver samples were analysed following the protocol adapted by Costas et al. [51] using the Pierce™ BCA Protein Assay Kit (Thermo Fisher Scientific, Waltham, MA, USA).

### 2.6. Data Analysis

The results presented below are expressed as mean ± standard error. All data were subject to statistical analysis by testing for homogeneity and normality of variances using Levene’s and Kolmogorov–Smirnov tests, respectively. To address skewed data on plasma cortisol, plasma and liver lactate and liver triglycerides, the data of these parameters were transformed logarithmically. Identification and comparison of significant differences in all parameters were performed by one-way ANOVA (*p*-value < 0.05) [57], followed by Tukey HSD post hoc test. Statistical analyses were conducted using IBM SPSS v25.0 (IBM Corp., Armonk, NY, USA).

## 3. Results

### 3.1. Cortisol

**Figure 2:**
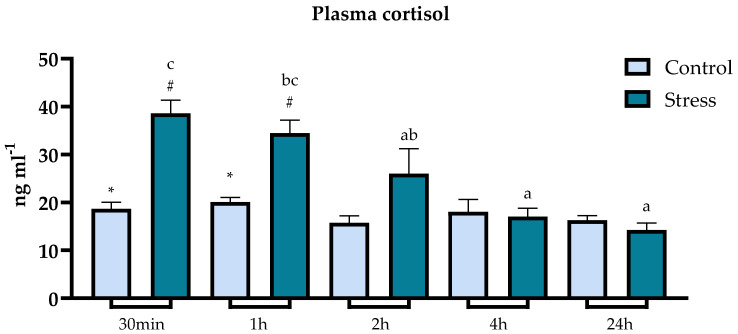
Plot showing cortisol levels (ng mL^−1^) in plasma (expressed as average ± SE) in undisturbed lumpfish (light blue bars) and in lumpfish exposed to air for 1 min (dark blue bars). Letters a, b and c indicate significant differences between different timepoints within the same group. Symbols * and # indicate significant differences between control and stressed groups within the same timepoint (ANOVA, Tukey, *p* < 0.05).

Plasma cortisol levels increased significantly in air-exposed fish compared to undisturbed counterparts, with the highest value found at 30 min post-stress exposure (38.61 ± 2.74 ng). Cortisol levels in stressed fish remained significantly elevated (ANOVA, *p* < 0.05) until 1 h after the acute stress exposure. Two hours after exposure cortisol returned to resting levels (Figure 2).

### 3.2. Metabolic Parameters

**Figure 3:**
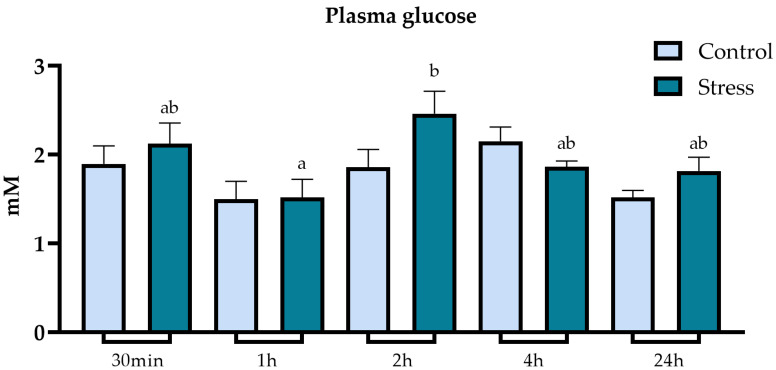
Plot showing plasma glucose (mM) levels (expressed as average ± SE) in undisturbed lumpfish (light blue bars) and in lumpfish exposed to air for 1 min (dark blue bars). Letters a and b indicate significant differences between different timepoints within the same group (ANOVA, Tukey, *p* < 0.05). No significant differences were found between stress and control groups.

Glucose in the plasma of stressed and undisturbed lumpfish did not vary significantly among them for each sampling time (Figure 3). However, there were significant differences between different timepoints within the air-exposed group, with a considerable increase from 1 h to 2 h post-stress.

**Figure 4:**
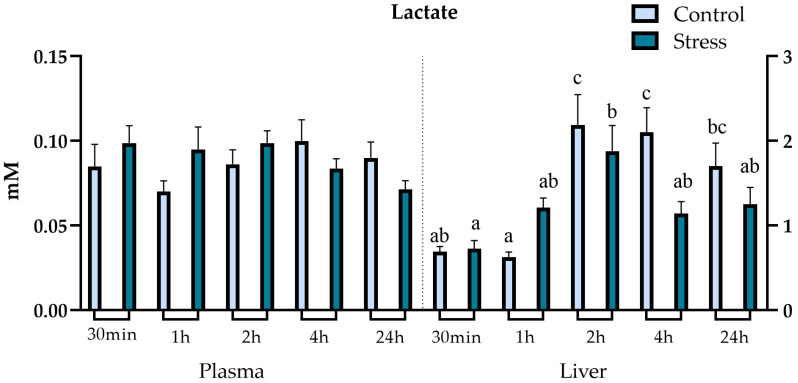
Plot showing plasma and liver lactate (mM) levels (expressed as average ± SE) in undisturbed lumpfish (light blue bars) and in lumpfish exposed to air for 1 min (dark blue bars). Letters a, b and c indicate significant differences between different timepoints within the same group (ANOVA, Tukey, *p* < 0.05). There were no significant differences between stress and control groups for both plasma and liver lactate.

There were no significant differences between control and stressed groups in both plasma and hepatic lactate (Figure 4) and total protein levels. However, there were significant differences in liver lactate levels between different timepoints within the air-exposed group, with a significant increase from 30 min to 2 h post-stressor, and within the control group, with a significant increase at 2 h. Liver lactate levels in undisturbed fish remained high from 2 h onwards.

**Figure 5:**
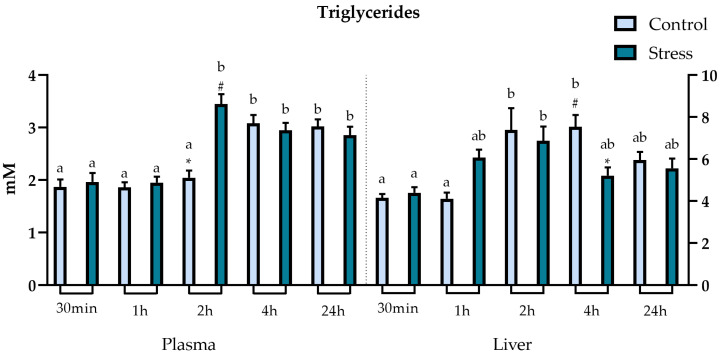
Plot showing plasma and liver triglycerides (mM) levels (expressed as average ± SE) in undisturbed lumpfish (light blue bars) and in lumpfish exposed to air for 1 min (dark blue bars). Letters a and b indicate significant differences between different timepoints within the same group. Symbols * and # indicate significant differences between control and stressed groups within the same timepoint (ANOVA, Tukey, *p* < 0.05).

Analysis of triglyceride levels revealed significant differences between stressed and undisturbed fish both in plasma and liver tissue (Figure 5). Regarding plasma triglycerides, stressed fish showed a significant increase in triglycerides at 2 h post-stressor, compared to undisturbed fish. There were also differences between timepoints within the same groups, where stressed groups increased at 2 h post-stress exposure, with the levels remaining high throughout all timepoints after that. The control group had a significant increase at 4 h and remained high at the last timepoint (24 h). Hepatic triglyceride levels were more elevated in undisturbed fish at 4 h post-stressor. There were also differences between different timepoints within the same groups. Both undisturbed and stressed groups displayed higher levels at the 2 h timepoint, remaining high in control groups during the 4 h timepoint.

### 3.3. Oxidative Stress

Oxidative stress analyses of acutely stressed fish and control groups are presented in Table 1. Fish exposed to acute stress had similar levels of catalase (CAT), superoxide dismutase (SOD) and lipid peroxidation (LPO) as the control groups. Oxidative stress was not affected by 1 min of air exposure. However, there were significant differences in both CAT and SOD throughout the 24 h time course in the control group. CAT activity was lower at 24 h compared to those sampled at 2 h, whereas SOD activity dropped its values at 24 h compared to 30 min and 4 h groups. LPO did not vary between stressed and undisturbed fish; however, it fluctuated throughout the 24 h sampling window within stressed fish, as well as in undisturbed animals, showing both groups’ highest LPO levels at 24 h post-air exposure.

## 4. Discussion

There is a need for reliable, cost-effective and technically accessible stress monitoring and profiling in lumpfish aquaculture. Besides the sub-optimal standardisation of practices in hatcheries, lumpfish must endure the industrial operations adapted for salmonids such as mechanical delousing, pumping, transfer and transportation and even slaughter [58,59]. Adapted for the salmon species, these processes are quite extreme and can be beyond the lumpfish’s ability to cope. Norwegian government authorities, research groups, academia and animal welfare-concerned aquaculture players have stressed the need for developing and implementing such practices [13,19].

The accuracy and usefulness of cortisol as a robust stress biomarker in teleosts are well documented [34,35]. In this study, air exposure for 1 min was sufficient to successfully activate the HPI axis. The acute stress challenge resulted in a detectable physiological response to stress, confirmed by the significant increase in cortisol levels. The measured cortisol peak at 30 min after exposure, which translates to a 107% increase in cortisol, indicates that the highest levels of cortisol happen between 30 min and 1 h following the stress exposure. The results are in line with the findings from other stress studies conducted with lumpfish, where cortisol peak was achieved between 30 min and 1 h after exposure [60,61]. For instance, a study where naive lumpfish (lumpfish that have never interacted with salmon) were introduced to Atlantic salmon detected a mean cortisol peak 45 min after stress exposure of 114.94 nmol L^−1^ (or 41.67 ng mL^−1^) [16], which is similar to the cortisol peak found in this study at 30 min following air exposure. Other studies also showed increased cortisol levels after acute stressor exposure using exhaustive chasing with a net [46], transport (76.3 ng mL^−1^ 1 h after exposure) [62] and crowding stress (50 ng mL^−1^ between 40 min and 1 h after exposure) [21]. Nevertheless, the levels recorded in this study were slightly lower than the peak achieved in the studies mentioned above. This may be attributed to the use of a milder stressor, which involved a brief and gentle netting, followed by 1 min air exposure. Selecting a severe stressor to achieve a marked stress response was not the scope of this study. So, following the refinement recommendations in the scope of PREPARE guidelines [63] inspired by the 3Rs [64] approach, we were able to confirm and study the activation of the HPI axis and subsequent secondary stress response using a less severe stressor.

Several physiological alterations occur due to the primary stress response activation. Cortisol and other stress hormones, such as catecholamines and β-endorphins, prompt measurable metabolic alterations during the secondary stress response. Metabolic biomarkers such as glucose and lactate levels vary as a result of intense exercise and severe stress. While glucose is a crucial source of energy in aerobic conditions, in anaerobic conditions the Krebs cycle is hampered due to lack of oxygen availability, and pyruvate is converted into lactate, accumulating in the muscle and bloodstream. Once oxygen is sufficiently available again, lactate can be transported to other organs such as the liver to be converted back into pyruvate for gluconeogenesis. Lactate can also play a role in helping to secure energy demands during aerobic metabolism [31,34,35]. The increase in glucose as a response to stress provides the fish with the energy substrate necessary to meet the elevated energy demands [65,66]. In this study, glucose and lactate levels in plasma did not differ significantly between stressed and undisturbed fish. These findings are in line with earlier investigations in lumpfish, where glucose and plasma lactate did not alter significantly. Lactate levels were barely detectable post-stress, even though a cortisol increase was observed, potentially indicating that lumpfish were less reliant on anaerobic metabolism [21,46]. However, there were significant differences within the stress group, with an increase of 2 h after exposure to stress. The increase in glucose 2 h post-stressor can potentially be related to the hyperglycaemic effects of cortisol, suggesting a lag effect of increased cortisol release on plasma glucose levels [35,67]. This is in agreement with previous reports in lumpfish, which did not observe any significant increase in plasma glucose following stress. The lack of significant effects on lactate in stressed fish has been reported for several other marine teleosts, which might indicate fundamentally different energy metabolism strategies in some fish species upon stress [29]. Furthermore, the cortisol effects on blood biochemistry and other components of the secondary stress response can be substantially different between different species of teleosts and depend on environmental factors [34,35]. Teleosts triglyceride reserves are stored in the liver, muscle and adipose tissues [68]. Mobilisation of triglycerides occurs as a response to the increased energy demands arising from stress. Free fatty acids are relevant energy substrates for teleosts and are released due to stress-promoted lipolysis of triglycerides [34,35,69]. However, the relation between triglyceride levels and stress in teleosts is not always straightforward and varies between teleost species and type of stressor, with studies reporting different variations in triglycerides in plasma and liver upon stress challenges [39,70,71,72,73]. This underscores the importance of understanding the species-specific effects and hypoxia tolerance. In this study, air exposure influenced the mobilisation of plasma and liver triglycerides, confirmed by the increase in plasma after 2 h and decrease in liver levels 4 h post-stress exposure. These results suggest that stressed lumpfish shifted the energy expenditure strategy to fatty acids as the preferred substrate.

The imbalance between the production and clearance of reactive oxygen species (ROS) can be designated as oxidative stress. Under normal circumstances, organisms are able to detoxify the reactive molecules and repair the oxidative damage using the organism’s antioxidant defences [74]. Upon stress exposure, the metabolic shifts necessary to meet the increased energy demands can lead to increased production of ROS, as a by-product of cellular respiration and other metabolic processes. Moreover, cortisol can modulate the expression and activity of antioxidant defences, such as superoxide dismutase, catalase and glutathione peroxidase [74,75]. This can affect the oxidative balance and contribute to the increased oxidative stress in the liver. Oxidative stress can contribute to oxidative damage, which can have consequences such as mutations in DNA, protein denaturation, lipid peroxidation and ultimately, cell death. The liver, being a crucial site for lipid metabolism, is susceptible to ROS damage by disruption of cell membranes, compromising hepatic cells and potentially impacting lipid metabolism [76]. In this study, the exposure of lumpfish to air for 1 min did not significantly alter the levels of SOD and CAT in the liver, when compared to undisturbed fish, despite a tendency for lower levels in stressed fish. This tendency could potentially be exacerbated with more severe acute stress exposure, as hypoxic conditions have been shown to modulate antioxidant defences [42,43,77]. In line with the results from SOD and CAT analyses, lipid peroxidation was not affected by stress, indicating that the oxidative stress in the liver of lumpfish is not affected by 1 min air exposure. While this study found no significant differences in oxidative stress, a prolonged, chronic exposure to stress could reveal a different outcome, highlighting the importance of further studies on the influence of chronic stress on lumpfish metabolic responses.

## 5. Conclusions

Lumpfish, a novel aquaculture species, has garnered attention due to the severe welfare concerns, and understanding species and stressor-specific physiological responses is an important step to help improve practices that safeguard lumpfish welfare. In this study, cortisol proved to be the most reliable indicator of stress in lumpfish exposed to a mild acute stressor, while glucose and lactate seem to be less valuable as stress biomarkers in lumpfish. Operations, such as salmon delousing, weighing and equipment maintenance, among others, could occasionally result in hypoxic conditions, as they often involve handling, capturing and regrouping of lumpfish. This information may be helpful for farmers in planning specific operations, where 1 min air exposure should not be surpassed, as this study showed that it is sufficient to activate a stress response while allowing a swift recovery.

## Figures and Tables

**Table 1:** Oxidative stress levels ^1^ in lumpfish liver homogenates.

Oxidative Stress	30minC	30minS	1hC	1hS	2hC	2hS	4hC	4hS	24hC	24hS
CAT	56.2 ± 4.3 ^ab^	55.2 ± 4.8	59.9 ± 2.7 ^ab^	64.4 ± 5.3	69.4 ± 7.1 ^b^	50.8 ± 2.4	63.9 ± 4.7 ^b^	52.0 ± 3.3	42.3 ± 2.4 ^a^	60.8 ± 4.5
SOD	2.0 ± 0.2 ^b^	2.0 ± 0.1	1.8 ± 0.1 ^ab^	1.7 ± 0.1	1.7 ± 0.2 ^ab^	1.4 ± 0.1	1.9 ± 0.1 ^b^	1.8 ± 0.1	1.3 ± 0.1 ^a^	1.5 ± 0.1
LPO	25.3 ± 1.5 ^b^	24.9 ± 1.2 ^bc^	20.7 ± 2.4 ^ab^	18.7 ± 1.4 ^a^	16.7 ± 0.8 ^a^	17.2 ± 1.2 ^a^	22.8 ± 1.3 ^ab^	26.0 ± 1.0 ^c^	32.5 ± 1.8 ^c^	29.7 ± 1.2 ^c^

^1^ Levels expressed as CAT (U mg protein^−1^), SOD (U mg protein^−1^) and LPO (tbars nmol g tissue^−1^) in undisturbed lumpfish “C” (light blue highlighted cells) and in lumpfish exposed to air for 1 min “S” (expressed as average ± SE). Letters a, b and c indicate significant differences between different timepoints within the same group (ANOVA, Tukey, *p* < 0.05). CAT: catalase; SOD: superoxide dismutase; LPO: lipid peroxidation.

## Data Availability

Data are contained within the article.
